# Model-Based Therapy Planning Allows Prediction of Haemodynamic Outcome after Aortic Valve Replacement

**DOI:** 10.1038/s41598-017-03693-x

**Published:** 2017-08-29

**Authors:** M. Kelm, L. Goubergrits, J. Bruening, P. Yevtushenko, J. F. Fernandes, S. H. Sündermann, F. Berger, V. Falk, T. Kuehne, S. Nordmeyer, CARDIOPROOF group, E. Morley-Fletcher, M. De Maldè, V. Muthurangu, A. Khushnood, M. Chinali, G. Pongiglione, A. Hennemuth, H. Mirzae, M. Neugebauer, O. Ecabert, D. Neumann, P. Groenenboom, G. Plank, D. Manset, A. McGuire, H. Naci, M. Salcher

**Affiliations:** 1 German Heart Centre Berlin, Department of Congenital Heart Disease, Unit of Cardiovascular Imaging, Berlin, Germany; 2 Charité – Universitätsmedizin Berlin, Biofluid Mechanics Laboratory, Berlin, Germany; 3 Charité – Universitätsmedizin Berlin, Department of Paediatric Cardiology, Berlin, Germany; 4 German Heart Centre Berlin, Department of Cardiothoracic and Vascular Surgery, Berlin, Germany; 5 DZHK (German Centre for Cardiovascular Research), Partner Site Berlin, Berlin, Germany; 6 Charité - Universitätsmedizin Berlin, Department of Cardiothoracic and Vascular Surgery, Berlin, Germany; 7 Institute for Computational and Imaging Science in Cardiovascular Medicine, Charité – Universitätsmedizin Berlin, Berlin, Germany; 8 Lynkeus Srl, Rome, Italy; 9 Institute for Cardiovascular Science, University College London, London, UK; 10 Department of Cardiology, Ospedale Pediatrico Bambino Gesù, Rome, Italy; 11 Cardiovascular Research & Development, Fraunhofer-Gesellschaft zur Förderung der Angewandten Forschung E.V., Bremen, Germany; 12 Image Analytics, Siemens Healthineers, Erlangen, Germany; 13 ESI Group, Paris, France; 14 Department of Biophysics, Medizinische Universität Graz, Graz, Austria; 15 Maat France Sarl, Argonay, France; 16 LSE Health, London School of Economics and Political Science, London, UK

## Abstract

Optimizing treatment planning is essential for advances in patient care and outcomes. Precisely tailored therapy for each patient remains a yearned-for goal. Cardiovascular modelling has the potential to simulate and predict the functional response before the actual intervention is performed. The objective of this study was to proof the validity of model-based prediction of haemodynamic outcome after aortic valve replacement. In a prospective study design virtual (model-based) treatment of the valve and the surrounding vasculature were performed alongside the actual surgical procedure (control group). The resulting predictions of anatomic and haemodynamic outcome based on information from magnetic resonance imaging before the procedure were compared to post-operative imaging assessment of the surgical control group in ten patients. Predicted vs. post-operative peak velocities across the valve were comparable (2.97 ± 1.12 vs. 2.68 ± 0.67 m/s; p = 0.362). In wall shear stress (17.3 ± 12.3 Pa vs. 16.7 ± 16.84 Pa; p = 0.803) and secondary flow degree (0.44 ± 0.32 vs. 0.49 ± 0.23; p = 0.277) significant linear correlations (p < 0.001) were found between predicted and post-operative outcomes. Between groups blood flow patterns showed good agreement (helicity p = 0.852, vorticity p = 0.185, eccentricity p = 0.333). Model-based therapy planning is able to accurately predict post-operative haemodynamics after aortic valve replacement. These validated virtual treatment procedures open up promising opportunities for individually targeted interventions.

## Introduction

Precisely tailored treatment remains an essential goal across several areas of medicine^1–3^. The ability to model, and thus to predict the functional response before the actual surgical procedure is performed holds great promise for improvements in heart valve surgery and has remained a yearned-for goal^4–7^.

Aortic valve disease (AVD) is the most common type of heart valve disease, with a broad set of treatment options and a steadily growing number of patients undergoing aortic valve replacement (AVR) and recurrent interventions^4,8^. Clinical guidelines provide the current standards for medical decision making such as indications for surgical valve interventions. They also highlight the need for reliable treatment procedures and heart valve types that re-establish physiologic haemodynamics^9,10^. Conversely, even after treatment most patients show abnormal blood flow patterns^5^. This is important because alterations in secondary flow and wall shear stress (WSS)^11,12^ can trigger endothelial cell dysfunction and wall remodelling of the aorta^13,14^. Abnormal flow patterns were shown to contribute to longer-term mortality and morbidity, including aortic aneurysm formation^15–17^, left ventricular remodelling and recurrent interventions^4,18,19^.

Valid prediction of post-operative haemodynamic outcome would be essential to identify the optimal type of treatment in a given anatomy. Computational fluid dynamics (CFD) has been used to gain additional information about haemodynamic parameters that are not directly assessable by clinical imaging technologies alone^20^. Imaging-based CFD was shown to provide valid functional information in a variety of cardiovascular diseases, offering additional guidance in the process of pre-treatment planning^21–23^.

However, a fully operational virtual valve treatment procedure requires the combination of several key elements and has therefore remained challenging and lacks clinical validation: (1) the patient-specific cardiovascular anatomy and (2) patient-specific flow characteristics need to be properly represented digitally; (3) virtual valve replacement procedure has to be performed in conjunction with (4) appropriate virtual modifications to the ascending aorta. Based on these patient-specific boundary conditions (5) CFD simulations are required and, finally (6), such individual model-based predictions need to be validated against the post-operative outcome after surgical AVR. In this study we aimed to combine these steps and validate the model-based prediction of haemodynamic outcome after AVR.

## Materials and Methods

### Study design and conduct

**Figure 1:**
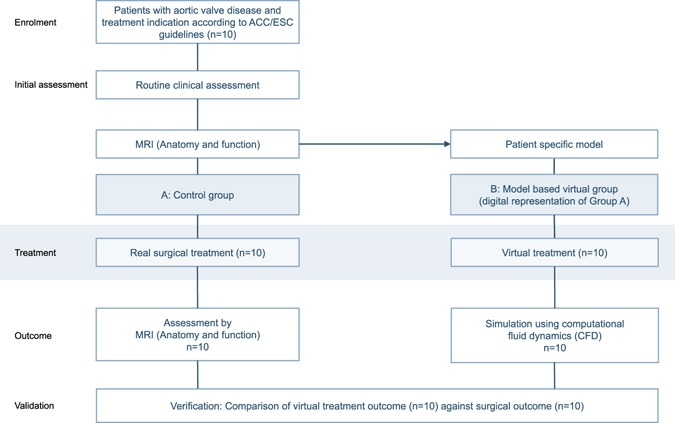
Design of the virtual intervention study. In parallel to the surgical treatment (Group A, control group) a virtual intervention was performed in a digital representation of the patients (Group B). Individual models were built in all patients. The predicted outcome after a virtual procedure was reassessed by computational fluid dynamics (CFD) and compared to the surgical control.

A virtual intervention study was performed in parallel to the actual surgical treatment (control group) using a single-case design in patients with AVD (Fig. 1). Ten patients with an indication for AVR with or without treatment of the ascending aorta were prospectively enrolled into the study meeting the inclusion criteria of the AVD study arm of the CARDIOPROOF trial. Patient characteristics are shown in Table 1. Table 2 summarises treatment information according to patient diagnosis. AVR typically includes open-heart surgery to replace the damaged native aortic valve with an artificial valve. The David procedure describes a method of aortic root replacement while sparing the aortic valve. During Ross procedure the diseased aortic valve is replaced with the patient’s own pulmonary valve.

**Table 1:** Baseline characteristics.

Characteristics	Patients (n = 10)
Age, median (range), years	51 (13–71)
Sex, no., m/f	9/1
Weight, median (range), kg	67 (55–98)
Height, median (range), cm	172 (162–185)
Body surface area, median (range), m²	1.8 (1.6–2.2)
Mean pressure across aortic valve, median (range), mmHg	36 (4–88)
Bicuspid aortic valve morphology, no. (%)	8 (80)
Aortic valve insufficiency, no. (%)	4 (40)
Aortic valve stenosis, no. (%)	6 (60)
Combined Aortic Valve lesion, no. (%)	2 (20)
Dilation of the ascending aorta, no. (%)	5 (50)
Ascending aorta Diameter, median (range), Z-score	3 (−3–9)
MRT-LVEF, median (range), %	63 (46–82)
MRT-LVEDV, median (range), ml/m²	79 (50–195)
Baseline systolic blood pressure, median (range), mmHg	138 (114–174)
Baseline diastolic blood pressure, median (range), mmHg	78.5 (45–100)
Baseline heart rate, median (range), bpm	71.5 (49–91)

LVEF = left ventricular ejection fraction, LVEDV = left ventricular end-diastolic volumes.

**Table 2:** Surgical treatment data.

Patient	Diagnoses	Valve treatment	Valve diameter	Treatment of the ascending aorta	Post MRI (days)
1	AI,AS,BAV	On-X Aortic(m)	23 mm	—	49
2	BAV,DA	David I procedure(b)	—	Hemashield, 30 mm	7
3	AI,BAV	Ross procedure(b)	—	—	174
4	AI,BAV,DA	SJM Masters HP(m)	25 mm	Hemashield, 32 mm	10
5	AS	CE Magna Ease(b)	21 mm	—	63
6	AS,BAV	CE Perimount(b)	25 mm	—	277
7	AS,BAV,DA	Medtronic HancockII(b)	21 mm	Reduction aortoplasty	137
8	AS,AI,BAV	Medtronic AVG(m)	21 mm	Hemashield, 24 mm	5
9	AS*,DA	SJM Regent repair(m)	23 mm	Hemashield, 28 mm	121
10	AS,BAV	Medtronic AP360(m)	20 mm	—	102

DA – dilated ascending aorta; BAV – bicuspid aortic valve; AI – aortic valve insufficiency; AS – aortic valve stenosis; (m) – mechanical valve; (b) – biological valve; SJM – St Jude Medical; CE – Carpentier Edwards, *stenosis of the mechanical valve.

All patients received a pre-operative magnetic resonance imaging (MRI) examination. At a median of 56 (5–277) days (see Table 2) after the operation all patients underwent a follow-up assessment, including post-treatment MRI. Using individual pre-treatment MRI data virtual valve replacement and model-based prediction of post-treatment haemodynamic outcome were performed in all patients, followed by a validation analysis comparing predicted and post-operative outcome.

The primary validation endpoints of the study were peak velocities across the valve and blood flow patterns across the valve (helicity, vorticity, and eccentricity). Secondary endpoints were wall shear stress and secondary flow degree.

The study was carried out according to the principles of the Declaration of Helsinki and approved by the local ethics committee (Ethics committee - Charité - Universitätsmedizin Berlin). Written informed consent was obtained from the participants and/or their legal guardians.

### MRI assessment

Cardiac MRI examinations were performed using a 1.5 Tesla Achieva R5.1.8. MRI scanner with a five-element cardiac phased-array coil (Philips Medical Systems, Best, The Netherlands). MRI protocols included routine three-dimensional anatomical imaging in end-diastole. The sequence parameters used were: acquired voxel 0.66 × 0.66 × 3.2 mm, reconstructed voxel 0.66 × 0.66 × 1.6 mm, repetition time 40 ms, echo time 2.0 ms, flip angle 90°, number of signal averages 3. Four-dimensional velocity-encoded MRI (4D VEC MRI) was used to capture flow data of the left ventricular outflow tract and the thoracic aorta (acquired voxel 2.5 × 2.5 × 2.5 mm, reconstructed voxel 1.7 × 1.7 × 2.5 mm, repetition time 3.5 msec, echo time 2.2 msec, flip angle 5°, 25 reconstructed cardiac phases, number of signal averages (1). Scan time varied between 9 and 14 minutes, depending on the size of the patient’s chest. High velocity encoding (3–6 m/s) in all three directions was used in order to avoid phase wraps in the presence of the valve stenosis or secondary flow. All flow measurements were completed with automatic correction of concomitant phase errors.

### Post-processing

Pre- and post-treatment anatomical MRI image data (3D MRI) of the aortic arch including the left ventricular outflow tract (LVOT) were segmented and reconstructed using ZIBAmira 2013.55 (Zuse Institute Berlin, Berlin, Germany) according to previous descriptions^12^. For qualitative visualisation and quantitative measurements of blood flow patterns within the ascending aorta 4D VEC MRI data analysis was performed using Gyrotools Flow (Release 2.2.15, Gyrotools, Zurich, Switzerland). Scalar maps (through-plane flow analysis) for grading of eccentricity were performed at three levels along the ascending aorta. Measurement at level 1 was performed just above the aortic valve, at level 2 caudally and at level 3 cranially to the pulmonary artery bifurcation. Aliasing was excluded in every image plane used for blood flow quantification in 4D VEC MRI. In order to use the personalised three-dimensional LVOT inflow velocity profile as inlet boundary condition for flow modelling, data were extracted from 4D VEC MRI using MEVISFlow (version 10, Fraunhofer MEVIS, Bremen, Germany) and interpolation on to computational mesh used ZIBAmira.

### Virtual treatment

The reconstruction of the pre-treatment anatomy was used for the virtual valve replacement procedure. The valve region corresponding to the height of the valve prosthesis ring including a margin of 3 mm in both directions was subsequently cut out using ZIBAmira. Computer models of valve prostheses (mechanical or biological valve) were inserted into the valve area using Gambit 2.4.6 (Ansys, Inc., Canonsburg, PA, USA) and the resulting surface was smoothed and cleaned using ReMESH version 2.0 (IMATI, Genoa, Italy) and MeshLab version 1.3.3 (ISTI, Pisa, Italy), respectively.

The type and size of mechanical valve was modelled according to the valve used during actual surgical treatment (Table 2). Patient 9 was a special case with stenosis of the mechanical valve prosthesis by a thrombus, which prevented one of two leaflets to function adequately. During surgical procedure the thrombus was removed and the valve function (opening and closing) was re-established without further need for valve replacement. Consequently, we called this case valve “repair”. In case of the insertion of biological valves, models were generated using the Fisics-Incor valve (INCOR, Hospital das Clínicas, University of São Paulo, São Paulo, Brazil). This valve approximates the anatomy of a set of commonly used biological valve prostheses, e.g. Hancock II and Mosaic valves (both Medtronic, Dublin, Ireland), Trifecta valve (St. Jude Medical, Inc., Saint Paul, MN; USA) and Perimount valve series (Edwards Lifesciences, Irvine, CA, USA). All virtual valve geometries were available in the STL file format as valve surfaces with leaflets in the fully open position and are part of a digital valve data base. The biological valves were based on 3D optical scanning as described before^24^ and were scaled to fit the size of the valve prosthesis used, while the mechanical valve models were generated using computer aided design software (CAD) (SolidWorks Corp., Waltham, MA, USA). In cases of surgical treatment of the ascending aorta (e.g. by Hemashield prostheses, Boston Scientific, MA, USA), aortic geometry of CFD models was modified accordingly during the virtual treatment procedure. In case of David and Ross procedure we hypothesized an ideal aortic valve generated according to a mathematical procedure proposed by Labrosse *et al*.^25^. In one case the aortic root was virtually replaced by a virtual graft of constant diameter, according to a digital representation of a type I David procedure.

### Computational fluid dynamics (model-based predictions)

Computational fluid dynamics (CFD) uses a numerical approach to assess flows (pressure and velocity fields). Necessary boundary conditions for CFD include anatomical data as well as inflow and outflow conditions. These data were based on individual MRI imaging data from each patient. Besides the predictive simulation after virtual treatment CFD with consistent inlet and outlet boundary conditions was used to assess additional haemodynamic parameters of the post-operative aortic anatomy.

**Figure 2:**
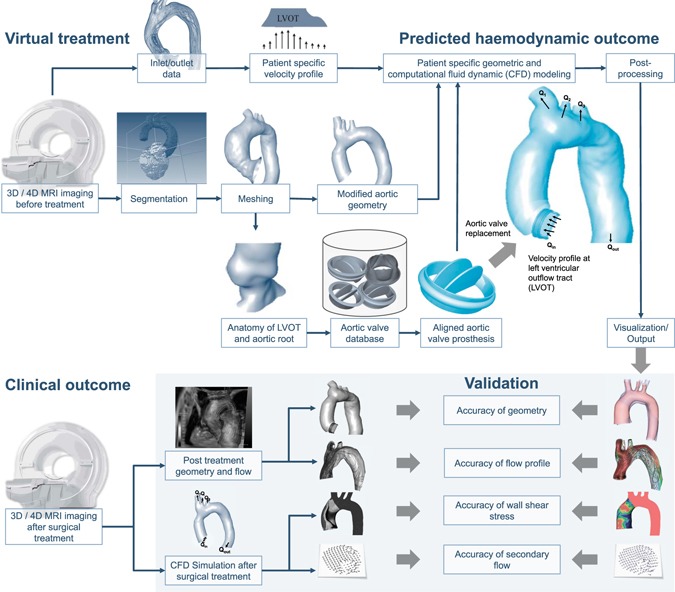
Virtual aortic valve replacement and clinical validation. The virtual aortic valve replacement procedure is outlined (upper panel of the figure). After virtual treatment a patient-specific computational fluid dynamic (CFD) simulation is performed, resulting in a prediction of the haemodynamic outcome. In the clinical validation process (lower panel of the figure) this outcome is compared against the clinical outcome of the patient.

Pre-treatment 4D VEC MRI data were used to set peak systolic flow conditions in the LVOT/ascending aorta and in the descending aorta. Flow rates in the head-neck vessels were calculated based on Murray’s law for the relation between flow rate and vessel diameter Q ~ d^3^ for branching vessels. Flow was simulated using ANSYS Fluent 14.5 (ANSYS Inc., Canonsburg, PA, USA) solving Navier-Stokes equations for the mass and momentum conservation laws. A non-Newtonian blood model was applied using an adapted power law model^26^. A k-ω SST transition turbulence model assuming constant turbulence intensity of 5% at the inlet surface was used. At the inlet of the simulated anatomy (LVOT) the velocity profile (three velocity components) was taken from pre-treatment 4D VEC MRI data and set for each node of the inlet boundary surface mesh using user defined function (programmed routine written in C). The procedure was described previously in the literature^23^. At all outlets the outlet boundary condition defining the flow rate at each outlet surface mesh applying zero diffusion flux for all flow variables and an overall mass balance correction was used. High-quality unstructured volume meshes accounting for approximately 5 million cells varying with the volume of the aorta were fabricated with the Gambit (ANSYS Inc.) following requirements and a mesh independence study for flow analysis in the aorta and aortic valve^23,24^. Convergence criteria were set to residual errors <10^−5^. The methodology of the virtual AVR validation study is summarized in Fig. 2.

### Comparison of anatomy between virtual and post-operative treatment

Anatomical differences were analysed by comparing averaged diameters of the ascending aorta as well as surface distances for aortic region without valve region. After alignment of the region of interest (common regions in both geometries) surface distances were calculated and quantified as mean surface distance with standard deviation and the Hausdorff distance (a measure of the maximal surface distance).

### Comparison between predicted and post-operative haemodynamic outcome

Predicted post-treatment blood flow patterns and velocities across the aortic valve were validated against the hemodynamic outcome after the actual surgical procedure. Based on CFD peak velocities were calculated and streamlines were visualised. These blood flow patterns were compared to measured 4D VEC MRI flow fields after surgical treatment. As described in recent clinical literature^5,6,13^ the comparison of helicity, vorticity and eccentricity of flow in the ascending aorta between post-operative 4D VEC MRI and CFD simulations was performed. Flow patterns in the ascending aorta were visualized using streamlines. Helical flow describes flow circulating along the longitudinal axis of the ascending aorta and vortical flow describes flow circulating along the vertical axis of the ascending aorta. Categorical grading was performed for describing the degree of helical and vortical flow profiles as follows: none = normal flow profiles, mild = flow rotation <360°, marked = flow rotation >360°. Eccentric flow was visualized using through plane velocity information in the ascending aorta. Grading was performed as follows: none = high velocity systolic flow was located in the centre of the vessel lumen, mild eccentric = high velocity systolic flow was not located centrally and was present in one- to two-thirds of the vessel lumen, marked eccentric = high velocity systolic flow is present along the rim of the vessel wall only (<one-third of the vessel lumen).

Secondary flow degree (SFD) is defined as the relationship between plane-averaged in-plane velocities to the through-plane velocity magnitudes^23^ and can be obtained from CFD simulations. In an additional validation step SFD was calculated based on predicted CFD, and compared to a CFD simulation based on MRI measurements after surgical treatment. The same was done to assess wall shear stress (WSS). In addition to surface-averaged WSS (WSS_SA_) we also analysed WSS based on the Hagen-Poiseuille law (WSS_HP_). WSS_HP_ is defined as WSS in a pipe with laminar full-developed flow (parabolic velocity profile): WSS_HP_ = ƞ × 4 × V_mean_/R_mean_, where ƞ is the dynamic viscosity of the blood (3.5 × 10^−3^ Pa·s), V_mean_ is the mean forward (through-plane) velocity and R_mean_ is the mean radius of the ascending aorta. At the same volume flow rate WSS_SA_ increases as secondary flow (e.g. vorticity, helicity) increases, whereas, WSS_HP_ remains constant. The simplified WSS_HP_ parameter simultaneously illustrates the impact of errors in post-treatment aortic diameter prediction, which exists even in the absence of complex flow patterns, as well as the difference between real and idealized WSS caused by secondary flow patterns. WSS_SA_ and WSS_HP_ after virtual treatment were compared to post-surgical values. The validation process is illustrated in Fig. 2.

### Statistical analysis

Data are presented as means ± standard deviation, unless stated otherwise. All data was tested for normality using a Kolmogorov-Smirnov test. Normally distributed data were analysed using paired t-test to compare differences between the predicted and post-operative haemodynamic parameters. Correlations were assessed by linear regression analysis. Friedman test was used to evaluate differences in blood flow patterns (eccentricity, helicity, vorticity) between methods. SPSS version 21 (IBM Corp., Armonk, NY, USA) was used for statistical analysis. In this study, Bonferroni correction was used to adjust for multiple validation endpoints and differences were considered significant if p < 0.0083. Bland-Altman plots were plotted to describe differences of measures between post-operative and predicted outcome.

## Results

### Anatomy

The analysis of anatomical differences between virtual and post-operative geometry found no significant differences between averaged diameters of the ascending aorta (28.3 ± 5.7 mm vs. 29.6 ± 6.9 mm, t = 1.684, d.f. (degrees of freedom) = 9, p = 0.126). A significant (p < 0.001) linear correlation D_virt_ = 1.0484 × D_real_ with R^2^ = 0.893 was found.

**Figure 3:**
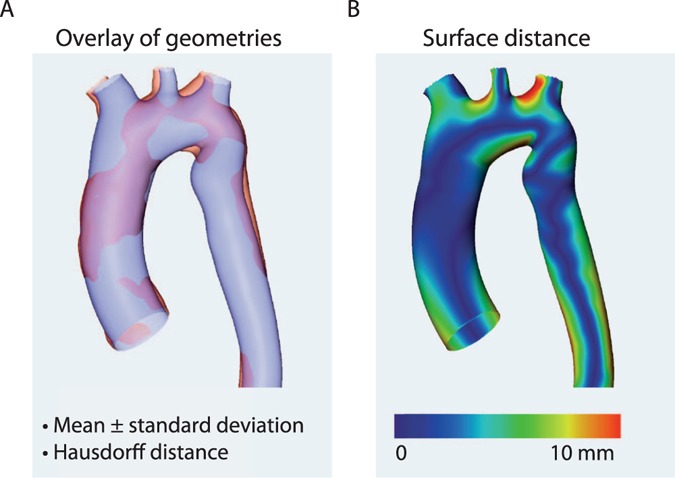
The comparison of aortic geometries (example). The accuracy of the vascular geometry is verified in order to ensure accurately set boundary conditions (**A**) The virtual anatomy is automatically overlaid with the actually resulting anatomy, providing mean differences between both measures and the Hausdorff distance. (**B**) shows the surfaces distance between geometries.

The averaged surface distance between two reconstructed ascending aorta surfaces for 10 cases was 1.9 ± 1.48 mm and therefore of the same order as the voxel resolution of MRI sequences used for the segmentation procedure. The averaged Hausdorff distance was 6.99 ± 4.21 mm (Fig. 3).

### Haemodynamic outcome

**Figure 4:**
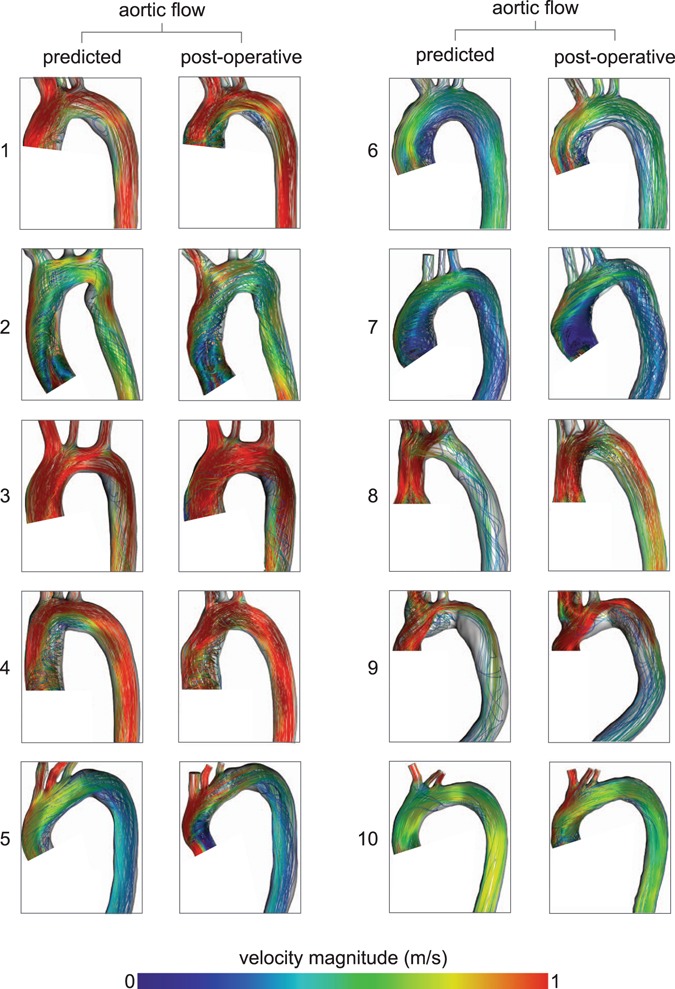
Visualization of flow patterns using CFD simulations based on post-operative imaging data (post-operative) and virtually treated preoperative imaging data (predicted).

**Figure 5:**
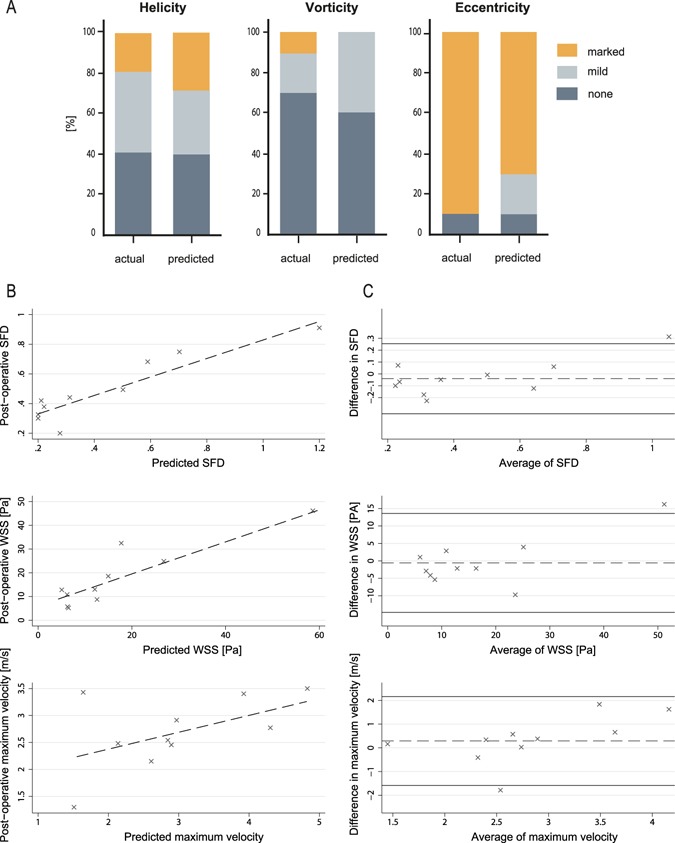
Comparison between predicted and post-operative haemodynamic outcome. (**A**) Comparison of blood flow profiles (helicity, vorticity, eccentricity, secondary flow degree) between the model-based prediction (CFD) and the post-operative flow profiles (4D Flow MRI) after a surgical procedure are compared. (**B**) Linear regression plots of predicted and post-operative secondary flow degree, surface averaged wall shear stress and maximal flow velocity across the valve. (**C**) Bland-Altman plots of predicted and post-operative secondary flow degree, surface averaged wall shear stress and maximal flow velocity across the valve, plotted against the mean. The continuous horizontal lines illustrate mean −1.96 and +1.96 standard deviations.

Predicted and post-operative blood flow profiles in the ascending aorta were comparable in all patients (Fig. 4). Categorical grading of helicity, vorticity and eccentricity of blood flow in the ascending aorta between post-operative and predicted outcome did not show significant differences (helicity p = 0.852, vorticity p = 0.185, eccentricity p = 0.333) (Fig. 5A). Maximal velocity across the aortic valve was not significantly different (2.97 ± 1.12 vs. 2.68 ± 0.67 m/s; t = 0.96, d.f. = 9, p = 0.362) and a significant (p < 0.001) linear correlation with R^2^ = 0.907 and a coefficient of 1.097 was found between predicted and post-operative maximal velocity magnitudes. Differences between predicted and the post-operative maximum velocities are visualized by Bland-Altman plots (Fig. 5D). CFD predicted transvalvular pressure drops across the virtual inserted valve prosthesis for the individual patients was as follows: (1–6.6 mmHg, 2–1.2 mmHg, 3–9.1 mmHg, 4–0.8 mmHg, 5–1.4 mmHg, 6–1.3 mmHg, 7–6.2 mmHg, 8–33.8 mmHg, 9–2.8 mmHg, 10–11.2 mmHg).

Comparison of secondary flow degree (SFD) in the ascending aorta between predicted and post-operative outcome found no significant differences (0.44 ± 0.32 vs. 0.49 ± 0.23; t = 1.156, d.f. = 9, p = 0.277) whereas significant (p < 0.001) linear correlation with R^2^ = 0.802 and a coefficient of 0.965 was found. The differences between predicted and post-operative SFD are visualised by Bland-Altman plots (Fig. 5B).

**Figure 6:**
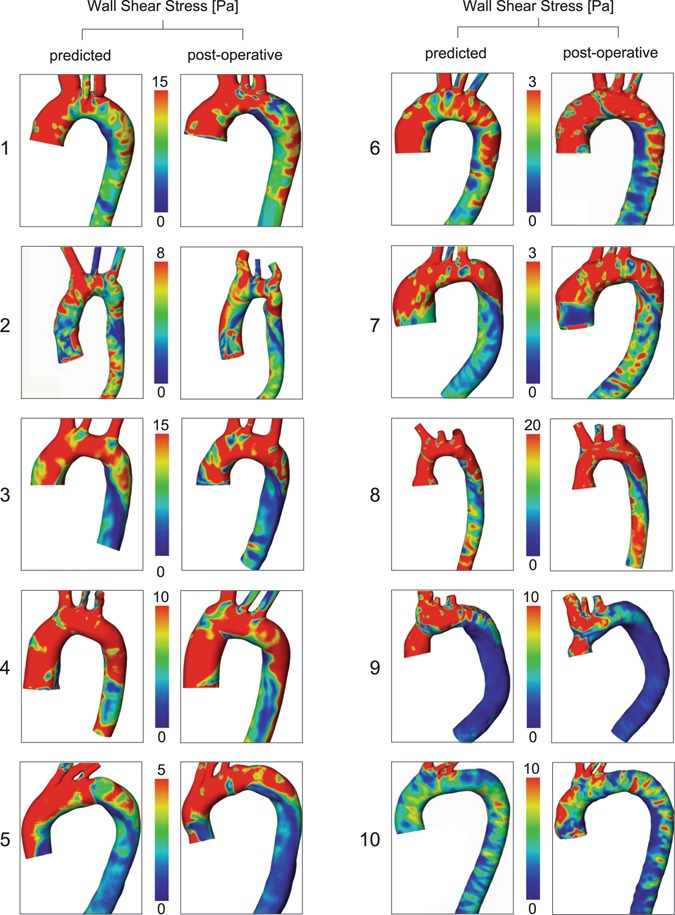
Visualization of WSS distributions using CFD simulations based on post-operative imaging data (post-operative) and virtually treated preoperative imaging data (predicted).

Hagen-Poiseuille based estimation of WSS in the ascending aorta in the predicted and post-operative treatment group showed comparable results (0.92 ± 0.91 Pa vs. 0.98 ± 0.96 Pa; t = 1.248, d.f. = 9, p = 0.243) with a significant linear correlation (WSS_HP,virt_ = 0.94 × WSS_HP,real_) (p < 0.001, R^2^ = 0.977). Similar results were found for WSS_SA_ values: no significant differences (17.3 ± 12.3 Pa vs. 16.7 ± 16.84 Pa; t = 0.259, d.f. = 9, p = 0.802), and a significant (p < 0.001) linear correlation with R^2^ = 0.821 and a coefficient of 1.06. The differences between predicted and the post-operative WSS are visualised by Bland-Altman plots (Fig. 5C). Individual WSS distributions are shown in Fig. 6.

## Discussion

The valid prediction of haemodynamics after aortic valve and vascular surgery opens up several opportunities for individualized pre-treatment planning, decision making and optimisation in patients with AVD. We were able to successfully perform the virtual procedure in ten patients with varying types of AVD and to use model-based therapy planning for accurate prediction of post-operative haemodynamic outcome after AVR. Thus, a fully operational virtual valve treatment procedure with all its challenges described was shown to be applicable in a first disease-specific study cohort of AVD patients.

Based on a fully non-invasive MRI assessment, treatment can be simulated prior to the actual procedure and entirely free of risk to the patient. Pursuing the aim of physiological flow condition restoration after AVR, the prediction of flow profiles can be helpful for decision making concerning (1) valve type, (2) size, (3) position, and (4) modifications to the ascending aorta. The avoidance of alterations in secondary flow and WSS can already help to limit several risk factors associated with long-term morbidity and mortality after AVR^16–18^. Furthermore, recent publications focusing on post-operative haemodynamic outcome after aortic valve surgery have shown significant differences between different types of valve prostheses^5,6,27^. In our patient population a high standard deviation for all examined parameters was observed, reflecting such high inter-individual variability after surgical treatment, as well as in its virtually predicted counterpart. While high variance is a strong motivator for improving treatment planning in the future it was accepted in the validation study as it replicates current clinical practice. There is still a marked eccentric flow pattern seen post-operatively in the majority of patients, which has been predicted correctly. The degree of eccentricity has been shown to be associated with the degree of growth rate of the ascending aorta in patients with bicuspid aortic valves^28^. Regarding WSS it has been described, that elevated localized WSS was associated with typical patterns of ascending aorta dilation^29^. In our patient population we have found regions of increased WSS leading to a higher value of averaged WSS along the ascending aorta. However, in order to possibly predict a none-eccentric flow pattern or a normalized WSS pattern along the ascending aorta, the validated procedure needs to be applied to patient specific geometries in combination with different types of devices or different surgical techniques^30^. The opportunity to test a diverse set of surgically implantable devices in a given patient-specific anatomy carries the potential for additional decision support regarding procedure types – potentially reducing the risk for recurrent interventions^31,32^.

In the majority of patients Bland-Altman plots showed sufficient agreement and regression plots showed good correlations for maximal velocity across the aortic valve, SFD and WSS in the ascending aorta, which already promotes confidence in the models’ explanatory power and outputs. The prediction of the maximal velocity across the aortic valve may play a role in individual prediction of prosthesis-patient mismatch, which has been described to be present in 19–70% of cases and was shown to impact mortality and morbidity after aortic valve replacement^33^. Maximal velocity can be converted to maximal gradient across the aortic valve and a difference of below 1.5 m/s would be a difference of mean gradient of approximately 4–5 mmHg, which was not considered to be a clinically relevant difference. The Bland-Altman plot shows only 1 patient with a value of >1.96 standard deviations for maximum velocity and show statistical comparability. Potentially, even lower differences could be of clinical relevance. However in clinical practice current imaging modalities used for assessing peak velocity (echo, MRI) also carry a similar risk for uncertainty in their measurements^34^. The clinical importance of differences found for surface averaged WSS values and SFD still need to be closer investigated in future clinical outcome-oriented studies. Nevertheless, in the literature WSS associated with normal endothelial function is defined as range between 1 and 15 Pa^35^ or even higher, the difference of 7–10 Pa would be considered as clinically acceptable. In 90% of the cases the Bland-Altman plot shows difference within this range. In regard to SFD it is described that elevation of SFD is present in the ascending aorta of patients with bicuspid aortic valves and associated with dilation of the ascending aorta^29^. In our 10 cases SFD varied between 0.2 and 1.2. In bicuspid aortic valve disease SFD of 2.5 has been observed^23^. Thus, differences of 0.2 we considered as clinically acceptable. In our patient cohort 80% of cases showed differences below 0.2.

The correctness of the prediction is limited by the accuracy of the boundary conditions. The personalisation of all simulations was achieved by adapting the model as closely as possible to individual anatomical and haemodynamic data. We used two anatomical parameters (averaged surface distance and Hausdorff distance) to verify the correctness of the geometrical boundary conditions. Instead of an idealized flow profile patient-specific LVOT-inflow profiles used were based on 4D MRI data assessed before treatment. By combining the patient’s pre-treatment imaging data with a database of aortic valve geometries a valid model of the interplay of LVOT specific flow and the open valve characteristics is provided, and was shown to correctly simulate the impact on post-operative flow in the ascending aorta^36^. As described previously^5,6^ we also observed disturbances in flow profiles and slightly elevated post-surgical flow velocities across biological and mechanical heart valves. As it was shown before we were able to confirm good agreement of blood flow patterns and velocity fields between 4D flow and CFD^37^. The present study contains data of a small patient cohort where the intent was to establish a virtual treatment workflow and validate its results in a first cohort of N = 10 patients. Further validation in larger cohorts will be required before considering its routine clinical application. Follow-up MRI were performed between 5–277 days after surgical treatment, thus, in few patients remodelling of the left ventricle could already have taken place and could have influenced the post-operative outcome parameters, which would not be predictable. However, good comparability of the predicted and post-operative parameters suggests that possible effects generated by remodelling processes might have only played a minor role. The predictive modelling in general is unable to take into account all possible changes in boundary conditions due to the real treatment procedure which can be considered a general limitation of all predictive models.

In a growing population of digitally represented patients the conduct of entirely virtual studies can be another prospect. Without any risk for the patient, inhomogeneity between treatment groups or carry-over bias, major challenges of interventional studies as well as conventional single case design trials could be overcome. In a virtual intervention cohort patients can safely undergo numerous different treatment methods under identical conditions.

Although cardiovascular models based on imaging data were demonstrated to produce results comparable to clinical diagnostic methods^22,37,38^, their combination with interventional study designs remained challenging and complex in heart valve disease. A technically and clinically oriented approach has recently helped to identify anatomical and functional target parameters in degenerative mitral valve disease with potential use for treatment planning^39^, and Kassab *et al*. recently highlighted the capabilities of new modelling technologies and quantitative approaches to surgical decision making^7^. It will, however, highly depend on the skills of the surgeon or interventional cardiologist to transfer such decisions to the patient. Additionally, haemodynamic outcome covers only one aspect of a treatment procedure. Several other non-haemodynamic problems can arise in patients undergoing AVR, affecting the overall morbidity and mortality^40^. Despite other risks nearly 50% of AVR procedures are linked to coronary artery bypass surgery^19^, representing an influence on the treatment group that has not yet been incorporated into the modelling simulations.

Future research should address these limitations in upcoming clinical trials. As aortic flow patterns were demonstrated to be consistent with post-operative patient data future in-silico studies will already be able to iteratively explore a variety of treatment options. The overall concept of a CFD-based virtual treatment and the validation methodology are not exclusively limited to AVD and could be of value in other structural heart valve disease, and in complex cases of congenital heart disease.

Computational modelling is desirable to bridge the gap between the existing situation and the emerging paradigms of precision medicine, in which patient-specific quantitative metrics and data guide therapy^7^. Such individually targeted interventions are likely to have an impact on clinical practice and the decision-making process. Our findings already support the potential benefit of clinical application of model-based predictions in aortic valve disease in near future. They open up the unprecedented opportunities for improvements in patient care, device selection, surgical strategy, and consequently the reduction of risk.
