# Publisher Correction: Model-Based Therapy Planning Allows Prediction of Haemodynamic Outcome after Aortic Valve Replacement

**DOI:** 10.1038/s41598-018-36022-x

**Published:** 2019-02-26

**Authors:** M. Kelm, L. Goubergrits, J. Bruening, P. Yevtushenko, J. F. Fernandes, S. H. Sündermann, F. Berger, V. Falk, T. Kuehne, S. Nordmeyer, CARDIOPROOF group, E. Morley-Fletcher, M. De Maldè, V. Muthurangu, A. Khushnood, M. Chinali, G. Pongiglione, A. Hennemuth, H. Mirzae, M. Neugebauer, O. Ecabert, D. Neumann, P. Groenenboom, G. Plank, D. Manset, A. McGuire, H. Naci, M. Salcher

**Affiliations:** 1 German Heart Centre Berlin, Department of Congenital Heart Disease, Unit of Cardiovascular Imaging, Berlin, Germany; 2 Charité – Universitätsmedizin Berlin, Biofluid Mechanics Laboratory, Berlin, Germany; 3 Charité –Universitätsmedizin Berlin, Department of Paediatric Cardiology, Berlin, Germany; 4 German Heart Centre Berlin, Department of Cardiothoracic and Vascular Surgery, Berlin, Germany; 5 DZHK (German Centre for Cardiovascular Research), Partner Site Berlin, Berlin, Germany; 6 Charité - Universitätsmedizin Berlin, Department of Cardiothoracic and Vascular Surgery, Berlin, Germany; 7 Institute for Computational and Imaging Science in Cardiovascular Medicine, Charité – Universitätsmedizin Berlin, Berlin, Germany; 8 Lynkeus Srl, Rome, Italy; 9 Institute for Cardiovascular Science, University College London, London, UK; 10 Department of Cardiology, Ospedale Pediatrico Bambino Gesù, Rome, Italy; 11 Cardiovascular Research & Development, Fraunhofer-Gesellschaft zur Förderung der Angewandten Forschung E.V., Bremen, Germany; 12 Image Analytics, Siemens Healthineers, Erlangen, Germany; 13 ESI Group, Paris, France; 14 Department of Biophysics, Medizinische Universität Graz, Graz, Austria; 15 Maat France Sarl, Argonay, France; 16 LSE Health, London School of Economics and Political Science, London, UK

Correction to: *Scientific Reports* 10.1038/s41598-017-03693-x, published online 29 August 2017

The original version of this Article contained a typographical error in the spelling of the author V. Falk, which was incorrectly given as Falk. This has now been corrected in the PDF and HTML versions of the Article.

